# Interferon for the treatment of genital warts: a systematic review

**DOI:** 10.1186/1471-2334-9-156

**Published:** 2009-09-21

**Authors:** Jin Yang, Yu-guo Pu, Zhong-ming Zeng, Zhi-jian Yu, Na Huang, Qi-wen Deng

**Affiliations:** 1 Department of Infectious Diseases, Nanshan Affiliated Hospital of Guangdong Medical College, Shenzhen 518052, PR China; 2 Institute of Dermatology and Venereology, Sichuan Academy of Medical Science & Sichuan Provincial People's Hospital, Chengdu 610031, PR China; 3 Department of Respiratory Diseases, West China Hospital of Sichuan University, Chengdu 610041, PR China

## Abstract

**Background:**

Interferon has been widely used in the treatment of genital warts for its immunomodulatory, antiproliferative and antiviral properties. Currently, no evidence that interferon improves the complete response rate or reduces the recurrence rate of genital warts has been generally provided. The aim of this review is to assess, from randomized control trials (RCTs), the efficacy and safety of interferon in curing genital warts.

**Methods:**

We searched Cochrane Sexually Transmitted Diseases Group's Trials Register (January, 2009), Cochrane Central Register of Controlled Trials (2009, issue 1), PubMed (1950-2009), EMBASE (1974-2009), Chinese Biomedical Literature Database (CBM) (1975-2009), China National Knowledge Infrastructure (CNKI) (1979-2009), VIP database (1989-2009), as well as reference lists of relevant studies. Two reviewers independently screened searched studies, extracted data and evaluated their methodological qualities. RevMan 4.2.8 software was used for meta-analysis

**Results:**

12 RCTs involving 1445 people were included. Among them, 7 studies demonstrated the complete response rate of locally-used interferon as compared to placebo for treating genital warts. Based on meta-analysis, the rate of Complete response of the two interventions differed significantly (locally-used interferon:44.4%; placebo:16.1%). The difference between the two groups had statistical significance (RR 2.68, 95% CI 1.79 to 4.02, P < 0.00001). 5 studies demonstrated the complete response rate of systemically-used interferon as compared to placebo for treating genital warts. Based on meta-analysis, the rate of Complete response of the two interventions had no perceivable discrepancy (systemically-used interferon:27.4%; placebo:26.4%). The difference between the two groups had no statistical significance (RR1.25, 95% CI 0.80 to 1.95, P > 0.05). 7 studies demonstrated the recurrence rate of interferon as compared to placebo for treating genital warts. Based on meta-analysis, the recurrence rate of the two interventions had no perceivable discrepancy(interferon 21.1%; placebo: 34.2%). The difference between the two groups had no statistical significance (RR0.56, 95% CI 0.27 to 1.18, P > 0.05). However, subgroup analysis showed that HPV-infected patients with locally administered interferon were less likely than those given placebo to relapse, but that no significant difference in relapse rates was observed between systemic and placebo. The reported adverse events of interferon were mostly mild and transient, which could be well tolerated.

**Conclusion:**

Interferon tends to be a fairly well-tolerated form of therapy. According to different routes of administration, locally-used interferon appears to be much more effective than both systemically-used interferon and placebo in either improving the complete response rate or reducing the recurrence rate for the treatment of genital warts.

## Background

### Description of the condition

Genital warts, which are also called condylomata acuminata or venereal warts, are the most common sexually transmitted disease (STD) in the general population[1]. The incidence of it is increasing rapidly and closely related human papillomaviruses(HPV) have been associated intimately with cervical neoplasia and other genital tract neoplasms [2-6]. It is estimated that 1% of sexually active people between the ages of 18 and 45 have genital warts. However, polymerase chain reaction (PCR) testing indicates that as many as 40% of sexually active adults carry HPV that causes genital warts. Genital warts are very contagious and could be spread during oral, genital, or anal sex with an infected partner. About two-thirds of people who have sexual contact with a partner with genital warts will develop warts, usually within three months of contact[7].

Genital warts vary somewhat in appearance. They may be either flat or resemble raspberries or cauliflower. The warts begin as small red or pink growths and grow as large as four inches across, interfering with intercourse and childbirth(in some cases). The warts grow in the moist tissues of the genital areas. In women, they occur on the external genitals and on the walls of the vagina and cervix; in men, they develop in the urethra and on the shaft of the penis.

Current treatment for genital warts is less than satisfying. No clear ideal therapy has been identified. Locally destruction methods, have mainly included surgical excision, electrocautery, cryosurgery and laser vaporization, which may result in scarring and are associated with recurrence. Chemical destructive methods using various acids, such as trichloroacetic or bichloroacetic acid, can be applied by the patients but are often locally irritating and not uniformly effective. Podophyllum resin, Podophyllotoxin, immune inducers (e.g., imiquimod), 5-fluorouracil cream can be used as a topical treatment. However, these medications require several weeks of treatment and may also irritate the skin [8-11].

### Decription of the intervention

In human body, Interferons are a class of small (15-28 kD) protein and glycoprotein cytokines (15-28 kD) produced by T cells, fibroblasts, and other cells in response to viral infection and other biologic and synthetic stimuli. IFNs bind to specific receptors on cell membranes. Their effects include inducing enzymes, suppressing cell proliferation, inhibiting viral proliferation, enhancing the phagocytic activity of macrophages, and augmenting the cytotoxic activity of T lymphocytes. As a curative drug, Interferon could be divided into three major classes (alpha, beta, gamma) on the basis of physicochemical properties, cells of origin, mode of induction, and antibody reactions[12].

Interferon has been shown to be active against HPV both in vitro and in vivo, to protect murine cells against infection with bovine papillomaviruses and to eliminate extrachromosomal viral DNA from infected cells [12,13]. The chief mechanism of its potential efficacy probably consists of following three fronts: (1) It acts as an antiviral agent; (2) it has an antiproliferative effect; and (3) it elicits an immune response from the host [13-16]. Based on these properties, interferon may lead to encouraging effects in the treatment of genital warts. It is reported that interferons exert their activities mainly by binding to specific membrane receptors on the cell surface. Once bound to the cell membrane, interferons initiate a complex sequence of intracellular events. In vitro studies demonstrated that these include the induction of certain enzymes, suppression of cell proliferation, immunomodulating activities such as enhancement of the phagocytic activity of macrophages and augmentation of the specific cytotoxicity of lymphocytes for target cells, and inhibition of virus replication in virus-infected cells.

By and large, interferon could be used either locally or systemically. Local administrations are mainly composed of intralesional injections and topical applications, in contrast to systemic administrations comprising subcutaneous and intramuscular injections.

### Why it is important to do this review

Due to the uncertainty of findings from current studies, what is more, HPV-infected patients must be provided with available information with which to make informed decisions regarding what kind of therapy for genital warts is relatively more effective, we aim to determine if there is any evidence from randomized controlled trials (RCTs) that the administration of interferon is efficacious for the treatment of genital warts.

## Methods

### Criteria for considering studies for this review

#### Types of studies

We included RCTs only.

#### Types of participants

HPV-infected patients. who were clinically or experimentally diagnosed as genital warts, were participated into our study.

#### Types of intervention

Interferon versus placebo. Interferon could be used either locally or systemically.

#### Types of outcome measures

##### Primary outcomes

Complete response rate and recurrence rate.

##### Secondary outcomes

Adverse effects such as flu-like symptoms(fever, chill, headache, myalgias, fatigue, etc), depression, anaemia, leukopenia, thrombocytopenia and so on.

#### Search methods for identification of studies

##### Electronic searches

We searched the electronic databases as follows: Cochrane Sexually Transmitted Diseases Group's Trials Register (January, 2009), Cochrane Central Register of Controlled Trials (the Cochrane Library 2009, issue 1), PubMed (1950 to 2009), EMBASE (1974 to 2009), Chinese Biomedical Literature Database (CBM) (1975 to 2009), China National Knowledge Infrastructure (CNKI) (1979 to 2009), VIP database (1989 to 2009). We also searched additional trials by scanning the reference lists of relevant trials identified. The search strategy was iterative as follows:

1 HPV

2 GENITAL WARTS

3 CONDYLOMATA ACUMINATA

4 VENEREAL WARTS

5 #2 OR #3 OR #4

6 INTERFERON

7 #1 AND #5 AND #6

8 (ANIMAL OR ANIMALS) NOT HUMAN

9 #7 NOT #8

##### Other search strategies

Organizations (including the World Health Organization), individual researchers working in the field were contacted in order to obtain possible additional references, unpublished trials, or ongoing trials, confidential reports and raw data of published trials.

##### Selection of studies

The titles, abstracts and keywords of every record retrieved were scanned to determine which were possibly relevant to the review. Any record that appeared likely to meet the inclusion criteria was obtained in full text. If there was any doubt regarding eligibility from the information given in the title and abstract, the full article was retrieved for clarification. Differences in opinion between reviewers were resolved by discussion.

##### Data extraction

Two review authors independently extracted data concerning details of the study population, interventions and outcomes using a standard data extraction form, specifically designed for this review. We resolved differences in data extraction by consensus, and with reference to the original article. If necessary, we sought information from the authors of the primary studies. For dichotomous outcomes, number of events and total number in each group were extracted. For continuous outcomes, mean, standard deviation and sample size of each group were extracted.

##### Assessment of risk of bias in included trials

The risk of bias was assessed based largely on the quality criteria specified by the Cochrane Handbook for Systematic Reviews of Interventions 5.0.1 [17]. In particular, the following factors were studied:

• Selection bias: a) was the randomization procedure adequate? b) was the allocation concealment adequate?

• Performance bias: were the patients and people performing the intervention blind to the intervention?

• Attrition bias: a) were withdrawals, dropouts and losses of follow-up completely described? b) was analysis performed by intention-to-treat?

• Detection bias: were outcome assessors blind to the intervention?

Based on these criteria, studies were broadly divided into the following three categories. This classification was used as the basis of a sensitivity analysis. Additionally, we intended to explore the influence of individual quality criteria in a sensitivity analysis.

• A: all quality criteria met - low risk of bias.

• B: one or more of the quality criteria only partly met -moderate risk of bias.

• C: one or more criteria not met - high risk of bias.

Each trial was assessed by two reviewers independently. Disagreements were resolved, where necessary, by recourse to a third reviewer. In cases of disagreement, the rest of the group were consulted and a judgment was made based on consensus.

##### Data Analysis

Statistical analysis was carried out by using Review Manager (version 4.2). Dichotomous data were presented as relative risk (RR) and continuous outcomes as weighted mean difference (WMD), both with 95% confidence intervals (CI). The overall effect was tested by using Z score with significance being set at P < 0.05. Heterogeneity was tested by using the chi-squared statistic and I square (I^2^) with significance being set at P < 0.1. Possible sources of heterogeneity were to be assessed by sensitivity and subgroup analyses. A fixed-effect model was to be used when the studies in the subgroup were sufficiently similar (P > 0.10, I^2 ^< 50%). A random effects model was to be used in the summary analysis when there was heterogeneity between the subgroups. Publication bias was to be tested by using the funnel plot or other corrective analytical method, depending on the number of clinical trials included in the systematic review.

## Results

### Description of studies

#### Studies identified

Our initial searches identified 1074 studies. No unpublished studies or other information was obtained from contact with WHO and individual researchers. By scanning titles and abstracts, 995 of them were excluded because they were duplicates, non-clinical studies, or had study objectives different from this review. After referring to full texts, 67 were excluded upon further scrutiny due to the following reasons: 42 studies were not real RCTs(of the 42 studies,17 were only case record analyses, and 25 did not develop a protocol before recruiting participants);16 studies had other interventions potentially impacting the outcome; 9 study had follow-up duration less than three months after the end of intervention with interferon. Finally, we included 12 studies, which involved a total of 1445 patients. Among them, 11 were published in English [18-28], 1 in Chinese(available only by searching the database of CNKI). Apart from Chinese and English, we did not search citations in other languages.

#### Designs of included studies

All the included studies were of a parallel design and had a control group.

#### Participants of included studies

Numbers of participants of the individual studies ranged from 42 to 257 with a total of 1445 participants included in this review. All of them were HPV-infected patients who were clinically or experimentally diagnosed as genital warts. The baseline characteristics (including the sex, age, race, mean lesion areas at study entry, median number of warts per patient, duration of diseases before therapy, and severity of disease, etc) were similar in the two groups (P > 0.05).

#### Interventions of included studies

Interferon used either locally or systemically was made as the intervention group, and placebo as the control group in each of the twelve studies.

#### Outcomes of included studies

The common outcome reported was the complete response rate and adverse events. However, of 12 included studies, only 7 reported recurrence rate.

### Methodological quality

#### Randomization

All the included studies were randomized controlled trials. Among them, 3 studies carried out the randomized assignment according to the randomization sequences generated by computer software.

#### Allocation concealment

Of 12 included studies, 1 study used sealed and opaque envelope to conceal the allocation process.

#### Blinding

Double blind was used in each included study. But there is lack of outcome assessor blinding.

#### Description of withdrawals, dropouts, losses of follow up and intention-to-treat analysis

2 of the included studies gave a description of losses of follow up and performed intention-to-treat analyses. 7 studies described withdrawals, dropouts or losses of follow up, but did not perform any intention-to-treat analysis. Other 3 studies did not describe any of them.

According to the quality criteria listed above, we considered all included studies were at moderate risk of bias and graded as category B

### Effects of interventions

#### Assessment of the Complete response rate of locally-used interferon versus placebo for treating genital warts

**Figure 1:**
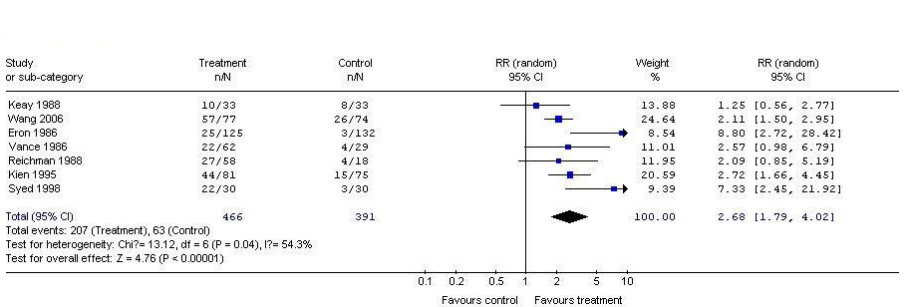
**Analysis of the Complete response rate of locally-used interferon versus placebo for treating genital warts**.

7 studies demonstrated the complete response rate of locally-used interferon (intralesional interferon or gel interferon) as compared to placebo for treating genital warts. According to chi-squared statistic and I square (I^2^), the results of the seven studies showed apparent statistical heterogeneity (p = 0.04.I^2 ^= 54.3%). So we used random effects model for meta-analysis. After synthesizing the results, we found out that the rate of Complete response of the two interventions differed significantly (locally-used interferon 44.4%; placebo: 16.1%). The difference between the two groups had statistical significance (RR2.68, 95% CI 1.79 to 4.02, P < 0.00001) (Figure 1).

#### Assessment of the Complete response rate of systemically-used interferon versus placebo for treating genital warts

**Figure 2:**
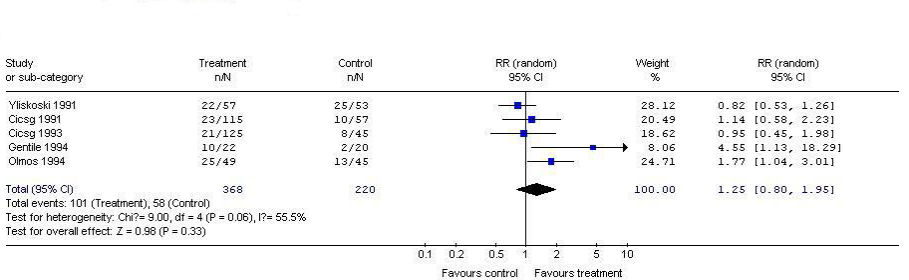
**Analysis of the Complete response rate of systemically-used interferon versus placebo for treating genital warts**.

5 studies demonstrated the complete response rate of systemically-used interferon as compared to placebo for treating genital warts. According to chi-squared statistic and I square (I^2^), the results of the 5 studies also showed apparent statistical heterogeneity (p = 0.06.I^2 ^= 55.5%). So we used random effects model for meta-analysis. After synthesizing the results, we found out that the rate of Complete response of the two interventions had no perceivable discrepancy (systemically-used interferon 27.4%; placebo: 26.4%). The difference between the two groups had no statistical significance (RR1.25, 95% CI 0.80 to 1.95, P > 0.05) (Figure 2).

#### Assessment of the recurrence rate of interferon versus placebo for treating genital warts

**Figure 3:**
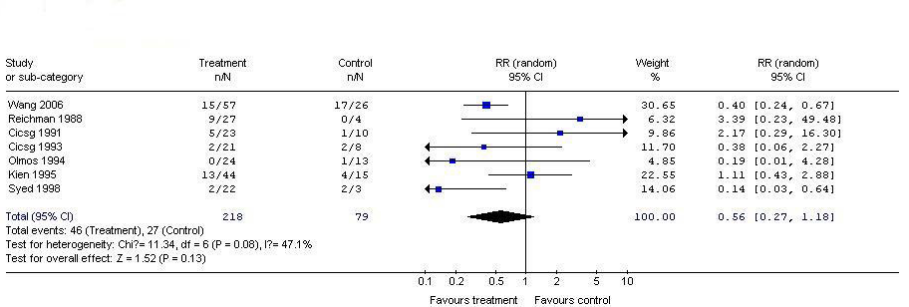
**Analysis of the recurrence rate of interferon versus placebo for treating genital warts**.

7 studies demonstrated the recurrence rate of interferon as compared to placebo for treating genital warts. According to chi-squared statistic and I square (I^2^), the results of the 7 studies also showed apparent statistical heterogeneity (p = 0.08.I^2 ^= 47.1%). So we used random effects model for meta-analysis. After synthesizing the results, we found out that the recurrence rate of the two interventions also had no perceivable discrepancy(interferon 21.1%; placebo: 34.2%). The difference between the two groups had no statistical significance (RR0.56, 95% CI 0.27 to 1.18, P > 0.05) (Figure 3).

#### Subgroup analyses

**Figure 4:**
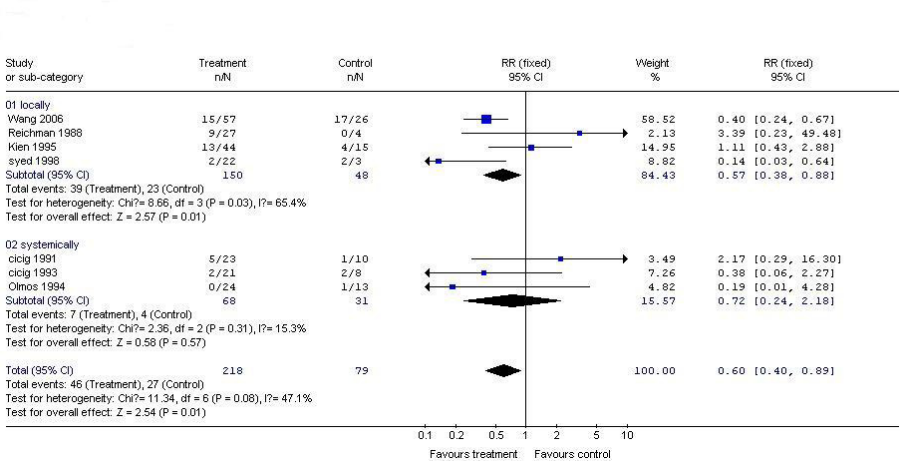
**Subgroup analysis of the recurrence rate of interferon versus placebo for treating genital warts**.

Of the 7 included studies that reported recurrence rate of interferon as compared to placebo for treating genital warts, 4 trials treated patients with locally-used interferon while 3 trails with systemically-used interferon. So subgroup analysis was carried out under the two circumstances. Results showed that the trend towards decreased recurrence rate of systemically-used interferon group compared to placebo group(RR 0.72, 95% CI 0.24 to 2.18) was lower than that of locally-used interferon group compared to placebo group(RR 0.57, 95% CI 0.38 to 0.88) (Figure 4).

#### Sensitivity analyses

We did not carry out any of the planned sensitivity analyses as no unpublished studies were found and all included studies were at similar risk of bias.

#### Assessment of publication bias

There was an insufficient number of trials for us to assess publication bias.

#### Adverse events

All included studies had reported the adverse events of interferon. The most common reported adverse reaction of systemic interferon was a flu-like syndrome, which was defined as the simultaneous occurrence of two of the following: fever/chills, headache, malaise/fatigue, and myalgia/muscle aches. Usually these flu-like syndromes were mild to moderate and decreased in incidence with repeated administration of interferon. Application-site reactions, such as itching, burning sensation and pain, occurred in parts of patients treated with intralesional interferon. In some trials, analysis of laboratory values obtained during and after systemic interferon therapy versus placebo revealed decrease in white-cell and platelet counts (leukopenia, thrombocytopenia). However, these values all returned to normal after the end of the treatment. There were no significant differences between the two groups in the results of liver-function tests (levels of serum aspartate aminotransferase, alanine aminotransferase) or in blood urea nitrogen or creatinine levels.

## Discussion

### Analysis of the effect of interferon for the treatment of genital warts

The immune status of the host is fairly crucial in the natural history of wart growth. This could be confirmed by the 20-30% rate of spontaneous remission during the first year of infection among otherwise healthy patients, and, on the other hand by the extensive refractory disease observed among immunosuppressed patients [28,29]. Conventional treatments for genital warts, including physical or chemical ablation of warts, may cause local issues such as inflammation, pain, ulceration or scars [30]. In addition, these methods only destroy visible lesions, but HPV may persist in normal-appearing epithelium adjacent to treated lesions, which results in a high rate of recurrences[8,31-33]. To the contrary, by way of its immunomodulating effect, interferon probably derives from its capacity to eradicate the virus from all the affected cells. For example, a T-helper lymphocyte deficiency, associated to an inversion of T4/T8 ratio has been observed in condylomatous lesions, with improvement of these conditions after interferon therapy[34].

In view of our study, 12 clinical trails were identified which evaluated the efficacy of interferon for the treatment of genital warts. As to complete response rate, they indicated locally-used interferon could achieve a clear beneficial effect while systemically-used interferon could not, both as compared to placebo. With regard to recurrence rate, interferon group appeared to show no lower recurrence rate than placebo group. However, by evaluating the included materials, we discovered that heterogeneity existed between the two groups. Hence, sub-group analysis was carried out under the two circumstances. The results demonstrated that HPV-infected patients with locally administered interferon were less likely than those given placebo to relapse, but that no significant difference in relapse rates was observed between systemic and placebo. As far as safety is concerned, interferon was not toxic and well tolerated, with a relatively low incidence of systemic adverse events, which were mild and did not require treatment interruption. In addition, one trial evaluated the different effects among three intralesional interferon preparations for treating genital warts, indicating that recipients of alpha-n1-, beta-, and alpha-2b-interferons had similar rates of complete resolution of lesions, although the member of patients enrolled in the study did not provide sufficient power to detect small but statistically significant differences among the different interferon preparations. In summary, there are at least two possible explanations for our observation that locally-used interferon could be more effective and achieve better long-lasting effects than systemically-used interferon. Because genital warts is widely regarded as a local illness, it is probable that warts are more sensitive to local administration, optimizing suppression of viral replication and cellular proliferation. Also, systemic administration of interferon may result in much lower intralesional effects of interferon. Thereby, locally-used interferon (intralesional injections or topical applications) are more worthy of recommendation than both systemically-used interferon (subcutaneous or intramuscular injections) and placebo for the treatment of genital warts.

Care should be taken, because both clearance rates and recurrence rates were measured at different times from the start of treatment in the 12 included trials(e.g.,4,8,16 weeks from baseline or different time duration from initial clearance). This may potentially influence the result of this review. However, currently, no standard time scales are available to measure clearance rate and recurrence rate in the treatment of genital warts. So it is difficult for us to make a proper inclusion criteria in study design. We believe that this point has also been considered by the authors of clinical trials. In light of it, we are expecting further research should provide standard time scales to better evaluate the clearance rate and recurrence rate of interferon therapy for genital warts.

### Limitations of this systematic review

Of 12 retrieved studies, only 3 trails describe randomization procedure. Other 9 did not give adequate descriptions of the methodology used. This may have misled us if we had not clarified the details, identifying the trials into category B rather than C. Allocation concealment is an important marker of trial quality. In a study of 250 controlled trials from 33 meta-analyses in pregnancy and childbirth, investigators found that alleged RCTs with inadequate and unclear allocation concealment yielded larger estimates of treatment effects (41% and 33%, respectively, on average)than trials in which authors reported adequate concealment[35]. However, very few potential articles considered for our review reported or performed allocation concealment, leading to high risk of selection bias.

Over and above, none of the studies mentioned blinding to the outcome assessors, which promotes suspicion of detection bias. In addition, 2 of the included studies gave a description of losses of follow up and performed intention-to-treat analyses. 7 studies described withdrawals, dropouts or losses of follow up, but did not perform any intention-to-treat analysis. Other 3 studies did not describe any of them. This may have led to relatively high attrition bias in our study. Otherwise, publication bias may exist as no primary articles reporting negative results were found.

### Implications for future researches

More high quality randomized controlled trials are required for assessing the effects of interferon for the treatment of genital warts. Especially, the randomization procedure should be clearly described, allocation concealment should be emphasized and the approaches should be reported. Besides, we expect that further detailed conducted, placebo-controlled studies of parenterally administered interferons should be carried out to examine the effects of different routes of administration, and more attention should be paid to combined treatment with interferon and other therapeutic agents on rates of regression and recurrence of genital warts.

## Conclusion

Interferon tends to be a fairly well-tolerated form of therapy. According to different routes of administration, locally-used interferon appears to be much more effective than both systemically-used interferon and placebo in either improving the complete response rate or reducing the recurrence rate for the treatment of genital warts.

## Competing interests

The authors declare that they have no competing interests.

## Authors' contributions

JY and YP equally conceived the study and made substantial contributions to its design, acquisition, analysis and interpretation of data. ZZ and ZY participants in the design, acquisition, analysis and interpretation of data. QD(corresponding author) participated in the design and revised the manuscript critically for important intellectual content. All authors gave final approval of the version to be submitted and any revised version.

## Pre-publication history

The pre-publication history for this paper can be accessed here:

http://www.biomedcentral.com/1471-2334/9/156/prepub
